# Strong interferon-gamma mediated cellular immunity to scrub typhus demonstrated using a novel whole cell antigen ELISpot assay in rhesus macaques and humans

**DOI:** 10.1371/journal.pntd.0005846

**Published:** 2017-09-11

**Authors:** Manutsanun Sumonwiriya, Daniel H. Paris, Piyanate Sunyakumthorn, Tippawan Anantatat, Kemajittra Jenjaroen, Suchintana Chumseng, Rawiwan Im-erbsin, Ampai Tanganuchitcharnchai, Suthatip Jintaworn, Stuart D. Blacksell, Fazle R. Chowdhury, Barbara Kronsteiner, Prapit Teparrukkul, Robin L. Burke, Eric D. Lombardini, Allen L. Richards, Carl J. Mason, James W. Jones, Nicholas P. J. Day, Susanna J. Dunachie

**Affiliations:** 1 Mahidol Oxford Tropical Medicine Research Unit, Mahidol University, Bangkok, Thailand; 2 Centre for Tropical Medicine and Global Health, Nuffield Department of Medicine, University of Oxford, Oxford, United Kingdom; 3 Department of Medicine, Swiss Tropical and Public Health Institute, Basel, Switzerland; 4 Faculty of Medicine, University of Basel, Basel, Switzerland; 5 Department of Veterinary Medicine, Armed Forces Research Institute of Medical Sciences (AFRIMS), Bangkok, Thailand; 6 Peter Medawar Building for Pathogen Research, University of Oxford, Oxford, United Kingdom; 7 Department of Medicine, Sappasithiprasong Hospital, Ubon Ratchathani, Thailand; 8 Department of Viral & Rickettsial Diseases, Naval Medical Research Center, Silver Spring, Maryland, United States of America; 9 Preventive Medicine and Biometrics Department, Uniformed Services University of the Health Sciences, Bethesda, Maryland, United States of America; 10 Department of Enteric Diseases, Armed Forces Research Institute of Medical Sciences (AFRIMS), Bangkok, Thailand; Institut Pasteur, FRANCE

## Abstract

Scrub typhus is a febrile infection caused by the obligate intracellular bacterium *Orientia tsutsugamushi*, which causes significant morbidity and mortality across the Asia-Pacific region. The control of this vector-borne disease is challenging due to humans being dead-end hosts, vertical maintenance of the pathogen in the vector itself, and a potentially large rodent reservoir of unclear significance, coupled with a lack of accurate diagnostic tests. Development of an effective vaccine is highly desirable. This however requires better characterization of the natural immune response of this neglected but important disease. Here we implement a novel IFN-γ ELISpot assay as a tool for studying *O*. *tsutsugamushi* induced cellular immune responses in an experimental scrub typhus rhesus macaque model and human populations. Whole cell antigen for *O*. *tsutsugamushi* (OT-WCA) was prepared by heat inactivation of Karp-strain bacteria. Rhesus macaques were infected intradermally with *O*. *tsutsugamushi*. Freshly isolated peripheral blood mononuclear cells (PBMC) from infected (n = 10) and uninfected animals (n = 5) were stimulated with OT-WCA, and IFN-γ secreting cells quantitated by ELISpot assay at five time points over 28 days. PBMC were then assayed from people in a scrub typhus-endemic region of Thailand (n = 105) and responses compared to those from a partially exposed population in a non-endemic region (n = 14), and to a naïve population in UK (n = 12). Mean results at Day 0 prior to *O*. *tsutsugamushi* infection were 12 (95% CI 0–25) and 15 (2–27) spot-forming cells (SFC)/10^6^ PBMC for infected and control macaques respectively. Strong *O*. *tsutsugamushi*-specific IFN-γ responses were seen post infection, with ELISpot responses 20-fold higher than baseline at Day 7 (mean 235, 95% CI 200–270 SFC/10^6^ PBMC), 105-fold higher at Day 14 (mean 1261, 95% CI 1,097–1,425 SFC/10^6^ PBMC), 125-fold higher at Day 21 (mean 1,498, 95% CI 1,496–1,500 SFC/10^6^ PBMC) and 118-fold higher at Day 28 (mean 1,416, 95% CI 1,306–1,527 SFC/10^6^ PBMC). No significant change was found in the control group at any time point compared to baseline. Humans from a scrub typhus endemic region of Thailand had mean responses of 189 (95% CI 88–290) SFC/10^6^ PBMC compared to mean responses of 40 (95% CI 9–71) SFC/10^6^ PBMC in people from a non-endemic region and 3 (95% CI 0–7) SFC/10^6^ PBMC in naïve controls. In summary, this highly sensitive assay will enable field immunogenicity studies and further characterization of the host response to *O*. *tsutsugamushi*, and provides a link between human and animal models to accelerate vaccine development.

## Author summary

Scrub typhus is a disease caused by bacteria that invade cells in our immune system and blood vessels. It is transmitted by mites and is treatable with antibiotics. Unfortunately diagnosis is difficult and requires techniques that are not easily accessible everywhere. Currently, there is no scrub typhus vaccine available. In order to improve diagnostics and vaccine development in future, we need to better understand our immune response against these bacteria. In this study, we developed a test where these bacteria were killed and prepared by a new purification method to stimulate the immune cells in our blood -not antibodies. We evaluated this test in hospitalized patients with scrub typhus disease and also in non-human primates to study the responses over time. The test proved to be very accurate and useful to study natural immune responses, and we found differences in responses in areas where scrub typhus is common, compared to areas where it is not common. This test will allow us to investigate the immune response to scrub typhus more in-depth in the future, and will support the development of better diagnostic tests and vaccines against scrub typhus.

## Introduction

Scrub typhus is a zoonotic illness caused by the intracellular bacterium *Orientia tsutsugamushi* which is endemic mainly across the Asia-Pacific region [1]. The pathogen is transmitted by the bite of larval Trombiculid mites known as chiggers [2, 3]. Scrub typhus is a febrile illness with a wide spectrum of disease severity from mild febrile illness to potentially fatal illness influenced by *O*. *tsutsugamushi* strains and host immune status. The specific skin lesion, known as an eschar, has been reported in up to 68% of Thai patients with scrub typhus [4]. The disease is treatable by antibiotics such as doxycycline, tetracycline or chloramphenicol [5], although emergence of antibiotic resistant strains has been reported in northern Thailand [6]. If untreated, the mortality is around 6% [7]. Awareness of and research into scrub typhus has been limited by its non-specific symptoms and difficulty of diagnosis, and a vaccine is highly desirable [8, 9].

A greater understanding of the host immune response to *O*. *tsutsugamushi* is required for vaccine design. As an obligate intracellular pathogen, cellular immunity is likely to be necessary for host control of infection. Several studies have reported a role for type 1 cell mediated immunity and specifically IFN-γ production in response to *O*. *tsutsugamushi* for immune protection against infection. Increased levels of IFN-γ and other type 1 cytokines are seen in the blood of patients with scrub typhus compared to controls [10–13]. Adoptive transfer experiments of monocyte-depleted splenocytes [14] and antigen-specific IFN-γ T-cells in murine models [15] have supported an important role for cell mediated immunity in protection against death from scrub typhus. Replication of *O*. *tsutsugamushi* inside macrophages is impaired by extrinsic IFN-γ [16]. *O*. *tsutsugamushi* infected monocyte-derived dendritic cells induce production of IFN-γ from CD4+ T-cells [17]. Scrub typhus is universally fatal in CD8-deficient mice (compared to 50% fatality in wild type mice) [18], and CD8+ T-cells play a vital protective role in control of *O*. *tsutsugamushi* growth [19]. In humans, CD8+ T-cell proliferation was seen during the convalescent phase of scrub typhus in patients [20]. These studies suggest the importance of developing a reliable method of monitoring *O*. *tsutsugamushi-*specific IFN-γ responses during scrub typhus.

The enzyme-linked immunospot (ELISpot) assay is a very sensitive technique allowing quantification of antigen-specific T-cells at the single cell level from peripheral blood by detection of IFN-γ or other secreted cytokines [21, 22]. The *ex-vivo* IFN-γ ELISpot assay is the most widely used technique for monitoring T-cell-based immune responses against intracellular pathogens such as HIV [23], tuberculosis [24] and malaria [25]. There are several advantages of the ELISpot assay for use in clinical trials: it has high sensitivity, is relatively easy to perform, uses low number of cells in the assay, does not require expensive instrumentation, and has the potential for high throughput screening with numerous specific peptides, or to an entire pathogen proteome using overlapping peptides of varying lengths. The ELISpot is up to 200 times more sensitive for cytokine detection than ELISA [26, 27] and significantly more sensitive than flow-cytometric based techniques [28].

Non-human primates (NHP) represent a model for investigating immunity to scrub typhus and provide valuable information to develop potential candidate vaccines for future testing in the clinical setting. Infection of cynomolgus macaques (*Macaca fascicularis*) with *O*. *tsutsugamushi* causes infection and illness which closely resemble the course of scrub typhus in humans [29, 30]. Due to some limitations of using cynomolgus macaques, such as reagent and antibody availability, the rhesus macaque (*Macaca mulatta*) is likely to be more suitable for preclinical vaccine evaluation.

In this study, we developed a novel *ex-vivo* IFN-γ ELISpot assay using whole cell antigen of *O*. *tsutsugamushi* (OT-WCA) as an antigen to determine magnitude and frequency of cellular responses in peripheral blood mononuclear cells (PBMC) of rhesus macaques. Our results indicate that our *ex-vivo* IFN-γ ELISpot assay can be used to determine immune responses against *O*. *tsutsugamushi* with high sensitivity and potentially high specificity for evaluation of vaccine candidate efficacy against *O*. *tsutsugamushi* in rhesus macaques and human clinical trials.

## Materials and methods

### Ethics statement

All animal research was performed strictly under approved IACUC protocol by the Institutional Animal Care and Use Committee and Biosafety Review Committee at the Armed Forces Research Institute of Medical Sciences (AFRIMS) Bangkok, Thailand, an AAALAC International-accredited facility. The IACUC protocol numbers are: PN12-01 (approved 31^st^ Jan 2012), and PN13-12 (approved 24^th^ Jan 2014). The animal research was conducted in compliance with Thai laws, the Animal Welfare Act, and all applicable U.S. Department of Agriculture, Office of Laboratory Animal Welfare and U.S. Department of Defense guidelines. All animal research adhered to the Guide for the Care and Use of Laboratory Animals, NRC Publication (8th Edition) [31].

Animals were housed individually in standard squeeze-type stainless steel cages with a minimum floor space of 4.4 square feet equipped with standard enrichments and exposed to ambient environmental conditions inside an Animal Biosafety Level 3 (ABSL-3) containment laboratory. Monkeys were fed daily with commercially prepared old-world primate extruded feed and supplemented with fresh fruit or vegetable four times per week. Fresh chlorinated water (5–10 ppm) was provided ad libitum via automatic water valves. Cages were cleaned daily and sanitized biweekly. All procedures were performed under anesthesia using ketamine hydrochloride, and all efforts were made to minimize stress, improve housing conditions, and to provide enrichment opportunities. Animals were euthanized by ketamine hydrochloride injection followed by barbiturate in accordance with the Guidelines for the Euthanasia of Animals (2013 Edition of the American Veterinary Medical Association).

The *O*. *tsutsugamushi* Karp strain (New Guinea) used in this study was provided by the Naval Medical Research Center (NMRC), Silver Spring, MD, USA and is a well-characterized strain from a pre-existing collection of Orientia strains at the NMRC, previously used in related studies [30, 32].

### Non-human primate model

Fifteen (9 male and 6 female) Indian-origin rhesus macaques (*M*. *mulatta* housed in AFRIMS (an AAALAC-accredited Program) were used in this study. Environmental conditions were maintained in accordance with the *Guide for the Care and Use of Laboratory Animals* 8^th^ edition (2011) [31]. The animals ranged from 3 to 5 year of age and weighed between 4.1 and 5.9 kg at the start of the study. The animals were evaluated to ensure that they were negative for SIV, SRV, STLV-1 and herpes B virus and had no *O*. *tsutsugamushi* exposure from the experimental history. Additionally, their antibody titers to *O*. *tsutsugamushi* were confirmed to be negative prior to the start of the experiment. Aliquots of inoculum containing defined concentrations of *O*. *tsutsugamushi* Karp strain at a dose of either 10^7^ or 10^7.8^ muLD_50_ (cultured and prepared in yolk sacs of chicken eggs), were used for intradermal (ID) injections of a total of 10 macaques. The inoculum was prepared in collaboration with the Mahidol-Oxford Tropical Medicine Research Unit (MORU) and the Naval Medical Research Center (NMRC), Silver Spring, Maryland, USA. The dosages were administered in infected groups on day 0. The inoculum was suspended in 100 μl of Snyder’s buffer and was applied to the anterior medial aspect of the left thigh. A total of 5 macaques were used as control group. The control macaques received ID injection with homogenized un-infected inocula in Snyder’s buffer at the identical site on the anterior medial aspect of the left thigh. Blood samples for PBMC isolation were collected at 5 time points starting from Day 0 prior to ID inoculation and every week up to Day 28. Bacteremia was determined using a previously describes qPCR assay for *O*. *tsutsugamushi*-specific 47 kDa gene [33]. DNA was extracted from 200 μl whole blood from each macaque using *DNeasy Blood & Tissue Kit* (Qiagen, Valencia, CA, USA) according to the manufacturer’s instructions. The *O*. *tsutsugamushi-*specific 47 kDa *htra* gene real time PCR assay was used as previously described using a CFX96 Real Time PCR Detection System (Biorad, Hercules, CA “no template” negative controls were run with each reaction and plasmid DNA served for standard curves in serial dilution from 10^6^ to 1 copies/μl of 47 kDa gene [34].

### Peripheral blood mononuclear cell (PBMC) isolation

PBMC were separated from 12 ml heparinized blood samples by density centrifugation within 3 hours of blood draw. In brief, 3 ml of Ficoll-HyPaque singular (Pharmacia, Peapack, NJ) was preloaded in a 14 ml LeucoSep tube (Greiner Bio-One) by centrifugation for 1 min at 1,000 × *g*. The whole blood was added to the LeucoSep tube and centrifuged for 15 min at 800 × *g* at room temperature. The cell suspension was collected, and the cells were washed twice in complete RPMI medium [RPMI 1640 (Sigma-Aldrich, St. Louis, MO) containing 10% FBS (Invitrogen Corp., Carlsbad, CA), 2 mM L-glutamine, 50 U/ml gentamicin (Quality Biological Inc., Gaithersburg, MD), and 0.1 mM non-essential amino acids (Sigma-Aldrich, St. Louis, MO)] for 5 min at 640 × *g* and 7 min at 470 × *g*, respectively. After final washing, the pellet was resuspended in complete RPMI medium before counting.

### Preparation of *O*. *tsutsugamushi* whole cell antigen (OT-WCA) for ELISpot assay

The *O*. *tsutsugamushi* Karp strain was cultivated in L929 cells (mouse fibroblast cell line) cultured in RPMI1640 supplemented with 10% fetal bovine serum (FBS) and 2 mM L-glutamine. The stage of infection was determined by indirect immunofluorescence assay (IFA); when infected L929 cells approached 100% positive infection, the cells were harvested by centrifugation at 6,000 x g for 30 min at 4°C. The cells were pelleted and disrupted using glass beads (0.1 mm, Next Advance, Averill Park, NY), then homogenized with a bullet blender for 1 min. After centrifugation at 300 x g for 10 min to remove cell debris, the supernatant was collected and filtered through a 2.0 μm syringe filter. The supernatant was then centrifuged at 11,000 x g for 10 min to collect the bacterial pellet. After washing the pellet with PBS, the bacteria were resuspended in 50 μl PBS and heated at 80°C for 1 hour. The OT-WCA suspension was then aliquoted and stored at 4°C until used, with immunogenicity of the antigen confirmed up to five years of storage. The total protein concentration of OT-WCA protein was determined by BCA assay (BCA1 kit, Sigma-Aldrich, St. Louis, MO). All processes were performed in a biosafety level 3 laboratory. The protein concentration of the stock solution for the OT-WCA used in this study was 1.41 mg/ml. Phytohemagglutinin (PHA) was used at a final concentration of 5 μg/ml and complete RPMI as described above was added to positive control wells and negative wells, respectively.

### *Ex-vivo* interferon-gamma (IFN-γ) enzyme-linked immunospot (ELISpot) assay

The kinetics and magnitude of the cellular responses to whole *O*. *tsutsugamushi* were assessed by *ex-vivo* IFN-γ ELISpot following an 18 hour stimulation of PBMC with OT-WCA for each time point of the study. Fresh PBMC were used in all ELISpot assays, and separate ELISpot kits (Mabtech, AB, Sweden) for human (3420-2A) and monkey (3421M-2A) cells were used. Briefly, 96-well Multiscreen-I plates (Millipore, UK) were coated for 3 hours with 10 μg/ml GZ-4 anti-human IFN-γ (Mabtech, AB, Sweden) at room temperature. Fresh PBMC were added in duplicate wells at 2x10^5^ PBMC in 50 μl per well and 50 μl of OT-WCA was added at the optimal concentration. For human studies, a T-cell epitope pool (Mabtech, AB, Sweden) at a final concentration of 1 μg/mL was used as control antigens. After 18 hours, secreted IFN-γ was detected by adding 1 μg/ml biotinylated mAb 7-B6-1-biotin for IFN-γ, which recognises an epitope completely conserved between human and macaques in the helical region of human IFN-γ, (Mabtech, AB, Sweden) for 3 hours and followed by 1 μg/ml streptavidin alkaline phosphatase (Mabtech, AB, Sweden). The plates were developed using the AP Conjugate Substrate Kit (Biorad, USA) according to the manufacturer’s instructions. ELISpot plates were scanned using a CTL ELISpot reader (Cellular Technology Limited, USA). Spots were then counted by Immunospot 3.1 software, using the manufacturer’s automated SmartCount^™^ settings. Results were expressed as IFN-γ spot-forming cells (SFC) per million PBMC. Background responses in unstimulated control wells were always less than 20 spots/10^6^ PBMC, and were subtracted from those measured in OT-WCA stimulated wells.

### Human samples

The recruitment of human subjects for immunological studies has been described previously in a study of melioidosis [35]. PBMC were isolated from subjects in Ubon Ratchathani, (northeastern Thailand—an endemic area for scrub typhus), Bangkok, (central Thailand—a non-endemic for scrub typhus) and Oxford, UK (non-endemic for scrub typhus) participating in a study of melioidosis. Responses to O. *tsutsugamushi* were evaluated using the same *ex vivo* IFN-γ ELISpot assay, with PBMC from Ubon Ratchathani subjects known to known to be reactive to OT-WCA used in Oxford as positive controls.

### Indirect immunofluorescence assay (IFA)

Human anti-*Orientia* antibodies (IgM/IgG) were detected using IFA for scrub typhus, based on pooled whole-cell antigens from three strains of *O*. *tsutsugamushi* (Karp, Kato and Gilliam strains) as previously described [36]. IFN-γ ELISpot responses were compared for people with IFA IgG titers of ≥ 1:400 compared to those with titres < 1:400.

### Statistical analysis

Statistical analyses were performed using GraphPad Prism Software v. 6. The results between the control group versus the infected group are expressed as means and were compared using the non-parametric Mann-Whitney U*-* test. Significant differences between timepoints within a group were determined with the non-parametric Wilcoxon t-test. The relationship between IFN-γ ELISpot responses and IFA IgG titers was evaluated using Spearman’s rank correlation test. Two-tailed P values < 0.05 were considered significant.

## Results

### Optimization of *O*. *tsutsugamushi* whole cell antigen (OT-WCA) concentration for use in an ex-vivo IFN-γ ELISpot assay

**Fig 1:**
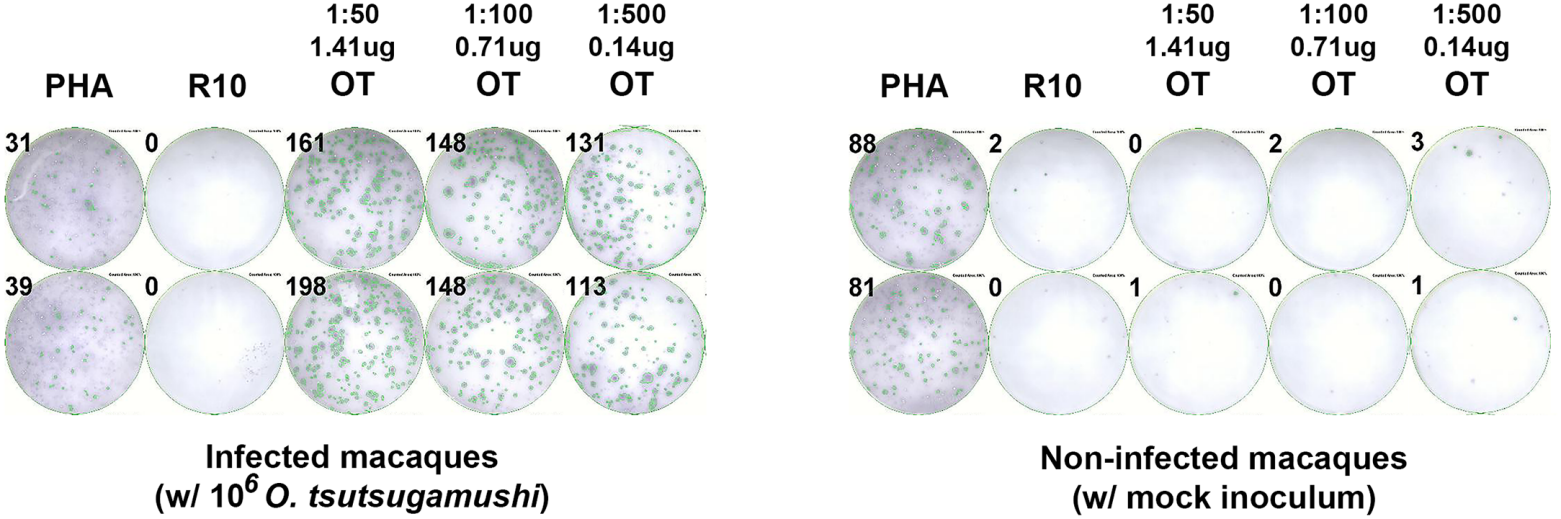
*Ex-vivo* IFN-γ ELISpot responses to *O*. *tsutsugamushi* whole-cell antigen (OT-WCA) using frozen PBMCs of rhesus macaques. Plate readout of *ex-vivo* IFN-γ ELISpot responses to OT-WCA from frozen PBMC of rhesus macaques 28 days after infection with 10^6^ *O*. *tsutsugamushi* (left) and non-infected control macaques (right) at 3 concentrations of 1.41 μg (dilution of 1:50), 0.71 μg (1:100) and 0.14μg (1:500) per well. PHA and ‘media’ are positive and negative control, respectively.

Frozen PBMC from rhesus macaques collected at Day 14 post inoculation (pi) with *O*. *tsutsugamushi* (BRI-02) and from a control uninfected macaque (BRI-06) were stimulated in duplicate with 50 μl of OT-WCA prepared at 3 different concentrations: 1.41 (1:50 dilution), 0.71 (1:100) and 0.14 (1:500) μg/well. PHA and ‘complete media’ were used as positive and negative controls respectively, (Fig 1 and Table 1). Strong IFN-γ ELISpot responses to OT-WCA were observed in infected macaques, whereas no responses to OT-WCA was observed in the uninfected macaque. Strong responses to PHA stimulation were found in positive control wells (Fig 1 and Table 1). Concentrations of OT-WCA above 1.41 μg/well were not tested because a blackout of the spot count was likely, and the optimized 0.14 μg/well concentration was selected for OT-WCA for the stimulation in further testing of experimental macaques.

**Table 1:** Optimization of the *ex vivo* IFN-γ ELISpot assay.

	Adjusted IFN-γ Spot Count (SFC/10^6^ PBMC)							
	OT infected				Controls			
	Well #1	Well #2	Mean	95%CI	Well #1	Well #2	Mean	95%CI
**PHA**	155	195	**175**	(0–429)	440	405	**422.5**	(200–645)
**Media**	0	0	**0**	(0–0)	10	0	**5**	(0–69)
**OT 1.41 μg**	805	990	**897.5**	(0–207)	0	5	**2.5**	(0–34)
**OT 0.705 μg**	740	740	**740**	(740–740)	10	0	**5**	(0–69)
**OT 0.141 μg**	655	565	**610**	(38–1182)	15	5	**10**	(0–34)

Note: Spot counts are expressed as spot forming cells per million peripheral blood mononuclear cells (SFC/10^6^ PBMC).

### Kinetics and magnitude of cellular immune responses over time with *ex-vivo* IFN-γ ELISpot

**Fig 2:**
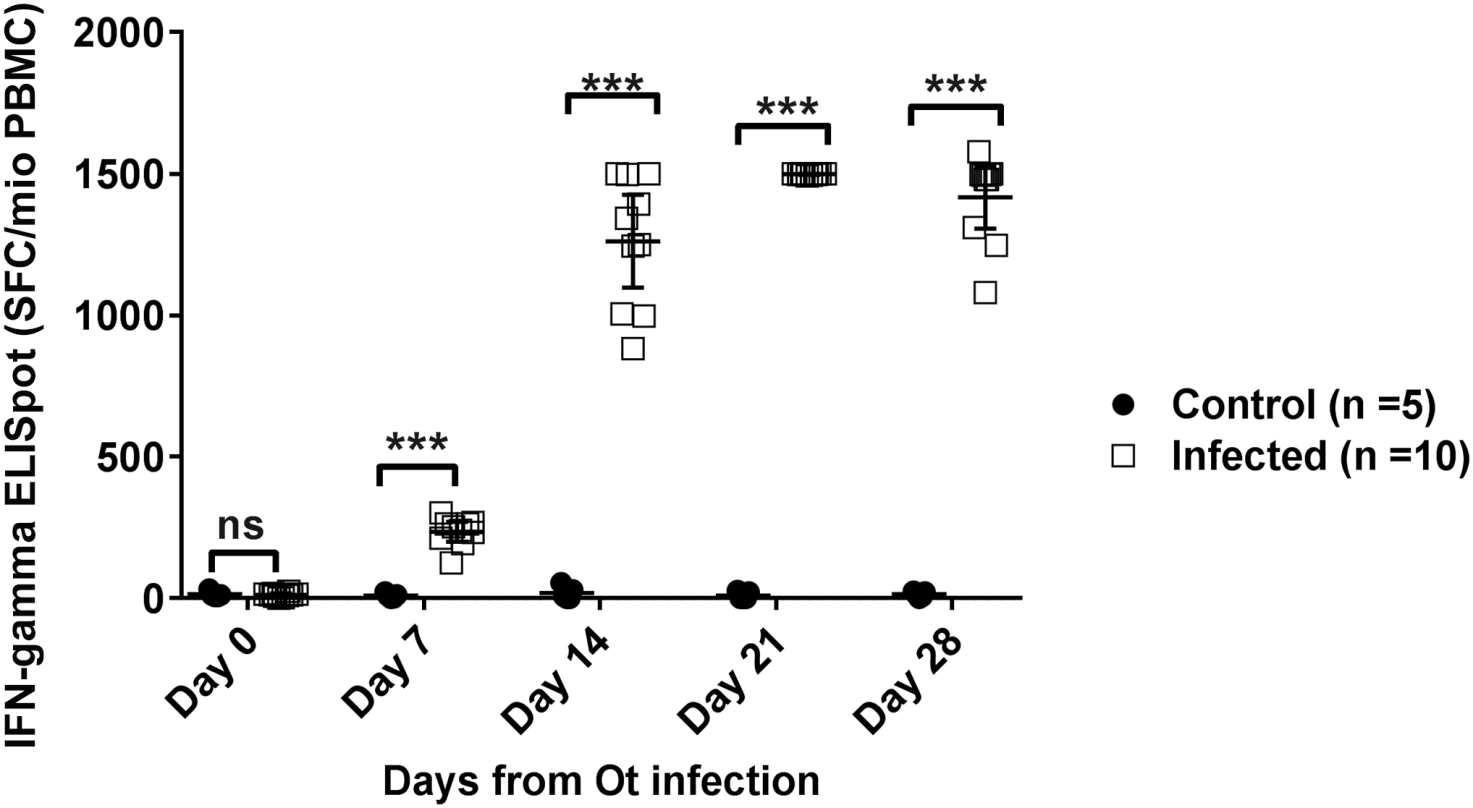
Cellular immune response kinetics by *ex-vivo* IFN-γ ELISpot. The kinetics of the cellular immune responses measured by *ex-vivo* IFN-γ ELISpot to OT-WCA after ID infection with *O*. *tsutsugamushi* (Infected, n = 10) versus mock injection (Control, n = 5) in rhesus macaques (panel A). *** = statistically significant difference (P ≤ 0.001) between Infected and Control groups by non-parametric Mann-Whitney *U-* test. Data plotted as means with 95% confidence intervals.

The *O*. *tsutsugamushi-*specific cellular immune responses were measured with *ex-vivo* IFN-γ ELISpot assay from freshly isolated PBMC of rhesus macaques at five time points after *O*. *tsutsugamushi* infection (Day 0, 7, 14, 21 and 28) to study the kinetics and magnitude of the responses over time (Fig 2). Freshly isolated PBMC from each macaque were tested in duplicate with OT-WCA at 0.14 μg/well. Each well contained 2x10^5^ PBMC, and SFCs were quantitated by the CTL ELISpot reader—therefore responses were multiplied by 5 to provide SFC / million PBMC. Strong responses were found from PHA stimulated wells in all macaques, and background responses assessed by media only were always less than 20 SFC/10^6^ PBMC (Table 2). Specific responses to *O*. *tsutsugamushi* were calculated by subtraction of corresponding media only wells from OT-WCA. Wells with very high responses resulted in blackout of the spot count and are represented as 300 spots (1,500 SFC/10^6^ PBMC), corresponding to the highest spot count that can be measured by CTL ELISpot reader in our experiment. Overall, *O*. *tsutsugamushi*-specific IFN-γ responses were observed from all infected macaques.

**Table 2:** Adjusted spot counts from *ex-vivo* IFN-γ ELISpot at each time point.

Adjusted spot count (SFC/10^6^ PBMC)																	
Days after infection	Control macaques (n = 5)						*O*. *tsutsugamushi* infected macaques (n = 10)										
	1	2	3	4	5	Mean	6	7	8	9	10	11	12	13	14	15	Mean
**Day 0**	7.5	12.5	7.5	12.5	32.5	**14.5**	0	15	2.5	12.5	10	12.5	7.5	17.5	15	25	**11.75**
**Day 7**	2.5	22.5	10	0	12.5	**9.5**	262.5	232.5	192.5	252.5	262.5	212.5	240	302.5	267.5	125	**235**
**Day 14**	7.5	30	0	0	55	**18.5**	1250	1392.5	1500	1500	997.5	1342.5	1245	1497.5	882.5	1005	**1261.25**
**Day 21**	0	27.5	0	0	22.5	**10**	1497.5	1500	1500	1495	1500	1500	1492.5	1497.5	1497.5	1500	**1498**
**Day 28**	22.5	25	7.5	15	0	**14**	1497.5	1500	1497.5	1577.5	1080	1480	1480	1310	1247.5	1495	**1416.5**

Values represent the mean SFC/10^6^ PBMC count from cellular immune responses to OT-WCA after subtraction of the background response (complete media) after ID administration of live *O*. *tsutsugamushi* (n = 10) versus mock (n = 5) in rhesus macaques.

At Day 7, the response to OT-WCA from infected macaques (mean 235 SFC/10^6^, 95% CI 200–270 SFC/10^6^) was 20-fold higher than baseline level (Day 0; mean 12 SFC/10^6^ and 95% CI 0–25 SFC/10^6^). At Day 14 the specific IFN-γ responses rose >100-fold (mean 1,261 95% CI 1,097–1,425 SFC/10^6^ PBMC), and were 125-fold higher at Day 21 (mean 1,498, 95% CI 1,496–1,500 SFC/10^6^ PBMC) and 118-fold higher at Day 28 (mean 1,416, 95% CI 1,306–1,527 SFC/10^6^ PBMC). No change in specific IFN-γ response to *O*. *tsutsugamushi* was found for uninfected control macaques. An overview of the adjusted spots count is shown in Table 2.

### Relationship between IFN-γ ELISpot response, IFA titer and bacteraemia in the non-human primate model

**Fig 3:**
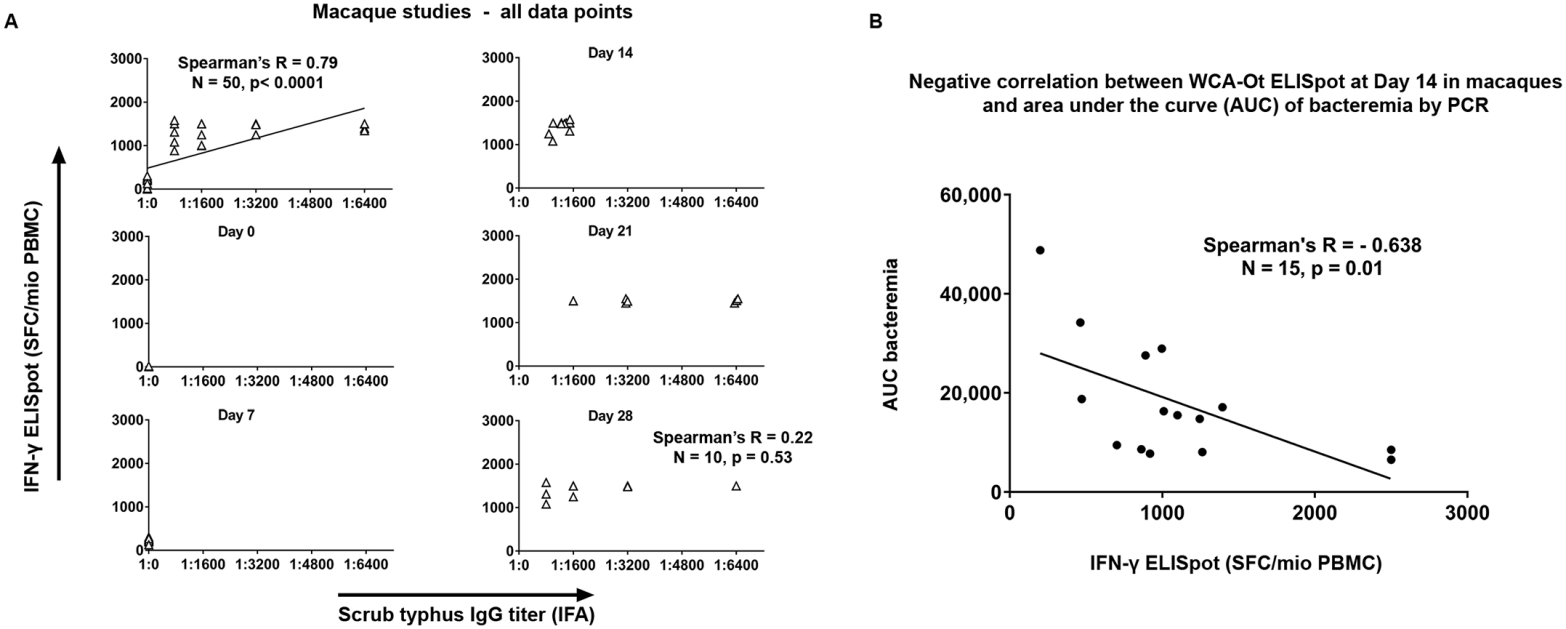
Relationship between the cellular response to *O*. *tsutsugamushi* in macaques measured by *ex vivo* IFN-γ ELISpot, the reciprocal titers of the IgG-based IFAs and bacteraemia. Panel A: The relationship between scrub typhus IgG antibody titer as determined by serum IFA reciprocal titers and cellular immune responses to OT-WCA antigen (as SFC /10^6^ PBMC) measured by *ex-vivo* IFN-γ ELISpot is shown for all 50 datapoints in the macaque model (top panel), and then separately for each timepoint (Day 0, 7, 14, 21 and 28). Scatter plot with linear regression line is plotted with Spearman’s R when significant. Panel B shows the negative correlation of the cellular immune responses to *O*. *tsutsugamushi* (SFC/10^6^ PBMC) and bacterial loads in blood (expressed as AUC of bacteremia) at Day 14, which corresponds to the peak bacteremia phase in this model.

We explored the relationship in the macaque model between *ex-vivo* IFN-γ ELISpot of cellular immune responses to *O*. *tsutsugamushi* and antibody responses to *O*. *tsutsugamushi* as measured by an IgG and IgM IFA assay. We saw a correlation between the magnitude of the cellular response and the reciprocal titers of the IgG-based IFAs in the non-human primate model (r 0.79 = P < 0.001 by Spearman’s rank test) (Fig 3, panel A). A significant correlation was not seen for each individual time point. This may be because there are only ten data points per timepoint and this is insufficient to make a correlation. In addition, the relationship in the first 28 days is limited by the IgG rising more slowly than the Elispot response, the latter plateauing by Day 21 (Fig 3, panel A), whilst IgG responses to infections are generally believed to peak later at around 4 to 6 weeks [37].

We also investigated the relationship of cellular immune responses to *O*. *tsutsugamushi* and bacterial load in blood (expressed as AUC of bacteremia) at Day 14, which corresponds to the peak bacteremia phase in this model, and found an inverse correlation, with increased SFCs /10^6^ PBMC relating to lower bacterial loads (Fig 3, panel B).

### Measurement by *ex-vivo* IFN-γ ELISpot of cellular immune responses to *O*. *tsutsugamushi* in human populations

**Fig 4:**
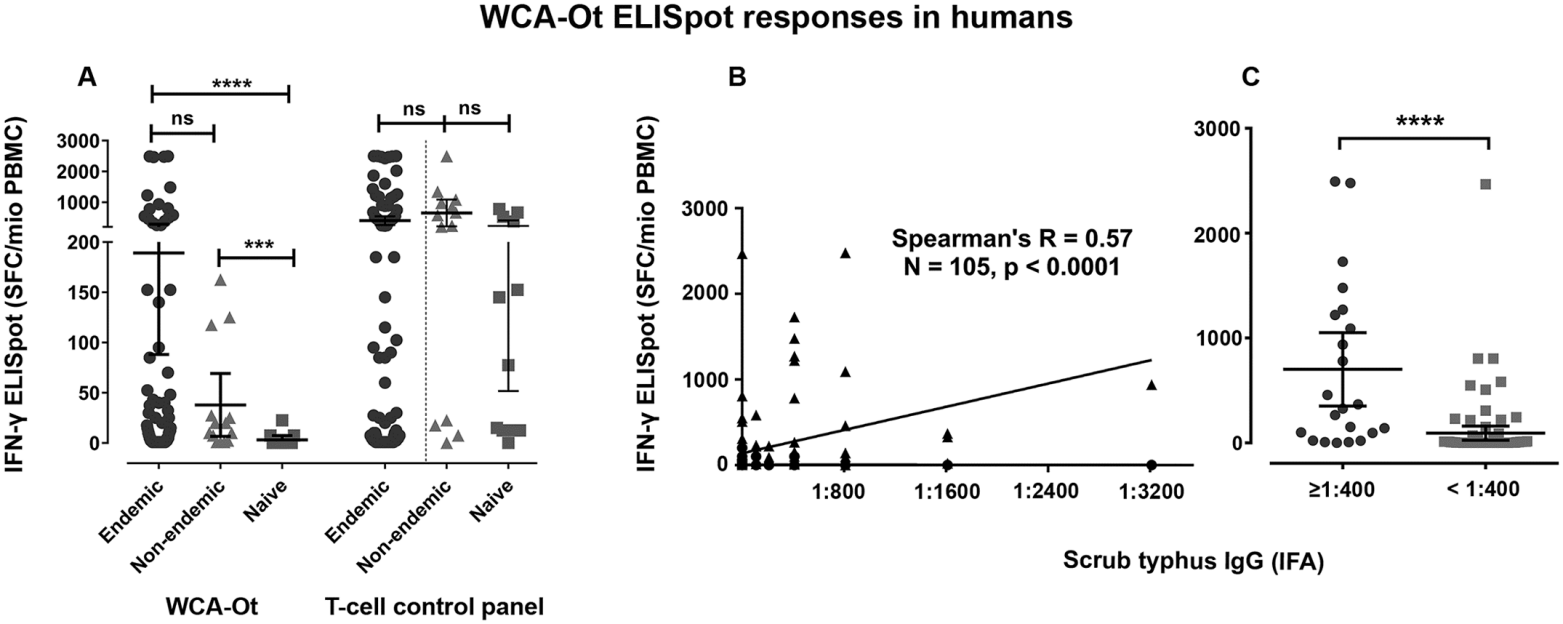
Human cellular immune responses measured by *ex vivo* IFN-γ ELISpot. Panel A shows human cellular immune responses measured by *ex vivo* IFN-γ ELISpot to OT-WCA and control panel of T-cell epitopes in people living in a region of Thailand endemic for scrub typhus (Ubon, n = 105), in a non-endemic area of Thailand where some of the population have had previous exposure (Bangkok, n = 14), and in the UK where there is no scrub typhus (Oxford, n = 12). Data is displayed as mean and 95% confidence intervals. Differences between groups were defined using the Mann-Whitney U test. **** P < 0.0001, *** P < 0.001, ns = not significant. Panel B shows the positive correlation of scrub typhus IgG antibody titer as determined by serum IFA reciprocal titers and cellular immune responses to OT-WCA antigen (as SFC /10^6^ PBMC) measured by *ex-vivo* IFN-γ ELISpot (n = 106). Scatter plot with linear regression line are plotted with Spearman’s R 0.57, p ≤0.0001. Panel C shows the comparison of cellular immune responses to OT-WCA antigen measured by *ex-vivo* IFN-γ ELISpot between people showing IgG IFA titers ≥ 1:400 (n = 22) and < 1:400 (n = 84). Data plotted with mean and 95% confidence intervals. Differences between groups assessed by Mann-Whitney U test.

In order to explore the feasibility of using this assay in human populations, responses to OT-WCA were measured in patients participating in a study of immune responses to a different disease (melioidosis). Subjects living in the scrub typhus endemic region of Ubon Ratchathani, Northeast Thailand (n = 105), had a mean IFN-γ ELISpot response of 189 SFC / 10^6^ PBMC (95% CI 88–290), compared to 40 SFC / 10^6^ PBMC (95% CI 9–71) in subjects living in a non scrub typhus endemic area (Bangkok, Thailand, n = 14) some of whom may have grown up in or travelled to an endemic part of Thailand, and 3 SFC / 10^6^ PBMC (95% CI 0–7) for subjects in Oxford, UK (n = 12) who had never encountered scrub typhus. 17/105 subjects (16%) in the endemic region had high responses greater than 200 SFC / 10^6^ PBMC compared to none in the non-endemic and naïve regions (Fig 4, panel A). No differences between groups were seen for responses to a control panel of common T-cell epitopes for Epstein–Barr virus (EBV), cytomegalovirus (CMV), influenza etc (“T-cell control panel”, Fig 4, panel A). Provisional studies showed that ELISpot counts were greatly reduced in responders if cryopreserved PBMC rather than fresh PBMC were used, suggesting a requirement for fresh antigen presenting cells to optimally process whole bacteria. As for the macaque model, we saw a correlation between the magnitude of the cellular response and the reciprocal titers of the IgG-based IFAs in the non-human primate model (Spearman’s R = 0.57, P < 0.001 by Spearman’s rank test) (Fig 4, panel B).

When cellular immune responses to OT-WCA antigen measured by *ex vivo* IFN-γ ELISpot were compared between humans with either an IFA IgG titer of 1:400 or above (n = 22) or less than 1:400 (n = 84), significantly higher cellular immune responses were found in people with the higher IFA (Fig 4, panel C).

## Discussion

We have established a highly sensitive method for measuring the magnitude and kinetics of the adaptive cellular immune response to scrub typhus in rhesus macaque monkeys. This study builds on previous work demonstrating production of IFN-γ in the host defense against *O*. *tsutsugamushi*. The OT-WCA ELISpot assay was developed using PBMC from rhesus macaques (*Macaca mulatta*), the most commonly used NHP model for preclinical vaccine development. To evaluate antigen-specific cellular responses to *Orientia* after infection with *O*. *tsutsugamushi* strain Karp, freshly isolated PBMC from a total of ten *O*. *tsutsugamushi* infected rhesus macaques and five uninfected control macaques were tested with the novel ELISpot assay from Day 0 through Day 28. *O*. *tsutsugamushi*-specific IFN-γ responses were observed post infection from all infected macaques compared to uninfected macaques, with a 20-fold (mean 235, 95% CI 200–270 SFC/10^6^ PBMC) increase at Day 7, a 100-fold increase at Day 14 and maintenance of high level responses to the end of the study (Day 28). The maximum measurable response was limited by the SmartCount^™^ software, which does not read any higher once a blackout of the well is obtained. No significant increases in cellular responses were found in the uninfected control group at any time point. Preliminary human studies in subjects from an endemic area, a non-endemic area where some of the population has previous exposure, and from non-exposed subjects gives support to the specificity of this assay for studying human populations, although the role of cross-reactivity to other rickettsial group pathogens merits further exploration.

The major reason for developing the IFN-γ ELISpot assay for *O*. *tsutsugamushi* is to allow immunogenicity monitoring in animal models, in scrub typhus exposed but healthy populations, in patient populations, and for future vaccine trials. IFN-γ was chosen as the read-out cytokine because of previous work demonstrating IFN-γ responses in scrub typhus patients [10–13] and mouse studies [14, 15]. The inverse relationship seen in this study for macaques 14 days post infection, where higher IFN- γ responses are associated with lower bacterial loads also lends support for the importance of the IFN-γ response in control of the bacteria, although other factors such as sicker animals having lower immune responses may be relevant. The importance of IFN-γ in response to intracellular pathogens has been reaffirmed recently by transcriptomic studies [38–40], and by a study demonstrating the link between BCG-specific T cells secreting IFN-γ and reduced risk of developing tuberculosis in South African infants [41]. However, other cytokine responses are important, for example IL-2 is involved in the development of memory responses and antibodies following vaccination against malaria [42], Hepatitis B [43] and tick-borne encephalitis [44]. Further studies by flow cytometry of multifunctional T-cell responses following scrub typhus infection are underway for humans and macaques.

For this initial characterization of the cellular response to *Orientia*, the OT-WCA was based on a single strain of high relevance in human disease (Karp strain).The antigen used for the IFA assay is based on pooled whole-cell antigens from three strains of *O*. *tsutsugamushi* (Karp, Kato and Gilliam) as this is the standard assay in the field. The conventionally used IFA slides based on the three reference strains have served over many years to document humoral responses against single strain *Orientia* infections, and results have been validated via gene sequencing methods [45, 46]. Ongoing work in the laboratory is exploring the immunogenicity of different strains and culture conditions.

A potential bias to the ELISpot assay is the potential for persistent presence of live *O*. *tsutsugamushi* bacteria in the PBMC of infected monkeys, driving the antigen specific response. However, we would expect to see responses in the media only in wells of the Day 7 and 14 macaque PBMC if residual bacteria were contributing to the measurable response. The cell culture media used contains penicillin and streptomycin to limit antimicrobial contamination in the laboratory, which may have a partial efficacy against the bacteria, but these antibiotics were used uniformly in all samples so should not introduce bias.

Previous vaccine development studies in cynomolgus macaques [29, 30] have demonstrated the presence of cellular immunity to two recombinant proteins derived from Karp stain *O*. *tsutsugamushi* (Kp r47b and Kp r56) using a 36-hour ELISpot assay. The magnitude of the IFN-γ responses to the whole bacteria reported in this manuscript was much higher than the levels to the individual proteins (range 125–800 SFC/10^6^ PBMC), alongside a lack of responses in unexposed animals. This is likely to be associated with the new and modified approach to purify *O*. *tsutsugamushi* to produce the OT-WCA suspension as described above. This OT-WCA ELISpot assay therefore allows better demonstration of cellular immunity to the whole bacteria, and can be performed as a positive control alongside exploration of immunity to specific vaccine candidate proteins such as Kp r47b and Kp r56. The magnitude of cellular immune response seen to OT-WCA compared to reported responses to the 47 and 56 kDa proteins suggest that there are other immunodominant antigens in *O*. *tsutsugamushi* for discovery as vaccine candidates. Stimulation with this preparation of killed *O*. *tsutsugamushi* in scrub typhus naïve monkeys did not invoke measurable innate cellular responses by this assay, for example from macrophages and NK cells via pattern recognition receptor pathways. This may be due to inactivated whole cell antigen being suboptimal at this dose for induction of innate pathways, or involvement of other key cytokines in pattern recognition, such as TNF-α, IL-12 and IL-1β not measured by this assay. In addition, potential evasion of the host innate immune response by *O*. *tsutsugamushi* is of interest in understanding the pathogenesis of natural infection and will be the subject of ongoing studies.

Scrub typhus diagnostics is a major difficulty for both management of patients and for epidemiological studies, and the lack of a clear-cut “gold standard” reference means that statistical modeling has been required to evaluate novel alternative diagnostic tests [36]. The OT-WCA IFN-γ ELISpot assay uses the same technology platform as the T-SPOT interferon-gamma release assay (IGRA) developed for diagnosis of tuberculosis [47]. Due to the laboratory processing requirements of ELISpot assays using fresh cells we do not believe there will be a role for using the OT-WCA IFN-γ ELISpot assay for real-time diagnosis and patient management of scrub typhus in endemic areas. However, given the unexpectedly high sensitivity and potential high specificity observed in this study, the OT-WCA IFN-γ ELISpot assay could potentially be used as a reference standard in research studies evaluating novel diagnostics. However evaluation regarding cross-reactivity with other rickettsial group bacteria and/or cross-protection in heterologous re-infection/infection in scrub typhus are needed.

The optimization of OT-WCA preparation for antigen stimulation studies provides a way of exploring the host response to *O*. *tsutsugamushi* by flow cytometry, thus allowing detailed characterization of cell phenotype, infected cells, secretion of cytokines and pathways of response to the bacteria. Immune-phenotyping studies of infected cells in eschar biopsies from scrub typhus patients have demonstrated a cellular tropism of *O*. *tsutsugamushi* for antigen presenting cells in the skin [48]. Although the ELISpot assay is a highly sensitive method for measuring cellular responses [21, 22], this method is unable to identify the cell phenotype secreting the cytokine. Further studies including a small volume, whole blood stimulation assay are underway to define which cells contribute most to IFN-γ secretion, and to characterize other cytokines associated with *O*. *tsutsugamushi* infection. This study did not address the cross-reactivity of immunity to Karp strain compared to other bacterial strains, and cross-reactivity with other rickettsial group bacteria in the region. Further studies are addressing this complex issue.

In summary, we have successfully developed for the first time a novel *ex vivo* IFN-γ ELISpot assay to whole *O*. *tsutsugamushi* antigen. This assay will allow field immunogenicity studies, pave the way for more detailed flow cytometry studies of response to *O*. *tsutsugamushi* antigen and provide a link between human and animal models to enhance vaccine development.
